# Cognitive Priming and Cognitive Training: Immediate and Far Transfer to Academic Skills in Children

**DOI:** 10.1038/srep32859

**Published:** 2016-09-12

**Authors:** Bruce E Wexler, Markus Iseli, Seth Leon, William Zaggle, Cynthia Rush, Annette Goodman, A. Esat Imal, Emily Bo

**Affiliations:** 1 Department of Psychiatry Yale University School of Medicine, 34 Park St, New Haven, CT 05619, USA; 2 National Center for Research on Evaluation, Standards, and Student Testing, CRESST/UCLA, 300 Charles E. Young Drive North, Los Angeles, CA, 90095USA; 3 C8 Sciences, 5 Science Park, New Haven, CT 06511, 970-371-1795, USA; 4 Department of Statistics, Yale University, 24 Hilhouse Ave, New Haven CT, 06511, USA.; 5 New Initiatives, 6 Cedar Lane, Cedarhurst, NY 11516, 917-584-0137, USA.

## Abstract

Cognitive operations are supported by dynamically reconfiguring neural systems that integrate processing components widely distributed throughout the brain. The inter-neuronal connections that constitute these systems are powerfully shaped by environmental input. We evaluated the ability of computer-presented brain training games done in school to harness this neuroplastic potential and improve learning in an overall study sample of 583 second-grade children. Doing a 5-minute brain-training game immediately before math or reading curricular content games increased performance on the curricular content games. Doing three 20-minute brain training sessions per week for four months increased gains on school-administered math and reading achievement tests compared to control classes tested at the same times without intervening brain training. These results provide evidence of cognitive priming with immediate effects on learning, and longer-term brain training with far-transfer or generalized effects on academic achievement.

Research in animals and humans has established that the structure and function of the brain are substantially shaped by environment-generated neuronal activity. To cite only three examples: auditory cortex of ferrets assumes the structure and function of the visual cortex when auditory input is surgically replaced by visual input^1^; targeted sensory stimulation and training in rats can reverse age-related changes in neuronal tuning, myelination and cytoarchitecture^2^; and practicing the violin for many hours as a child is associated with expansion of the right sensori-motor cortex opposite the left hand which makes rapid and complex finger movements^3^.

Medical scientists have created new treatments based on this neuroplastic potential. Most dramatic are sensory substitution devices that allow blind people to see. A camera worn as spectacles sends a television-like image to a small, dense grid placed on the tongue and the brain processes it as if the afferent input was from the eye^4^. Other researchers have used computer-presented neurocognitive exercises to produce activity-dependent enhancement of under-functioning neural systems in people with schizophrenia^5^,^6^ or depression^7^. Benefits generalized to non-practiced cognitive operations^8^, transferred to increased job success in the community a year after intervention^9^, normalized task-related regional brain activation^10^, and led to clinical recovery in patients who had failed to respond to medication^7^.

These basic science and clinical studies encouraged researchers and industry to create computer-presented brain-training games (BT) for children, when the brain is highly plastic, with goals of improving attention, other thinking abilities and academic achievement^11^. Recent reviews of some of this work, however, have concluded that the programs evaluated showed little or no benefit beyond improvement on the BT games themselves, or on very similar cognitive tests^12^,^13^. While the basic science and clinical evidence of neuroplasticity suggest that the limitation may be in the particular BT programs studied rather than the enterprise itself, research is needed to confirm that BT can help children, especially in the real world of school implementation.

We designed four types of computer-presented BT games to increase executive function in school children. Executive function is a suite of cognitive operations that are important in managing oneself and managing information, and includes focused attention, response inhibition, working memory and cognitive flexibility. Executive function skills when children are five to seven years old predict success throughout school^14^,^15^,^16^. The first of the four games used in the present study trains simple sustained attention at its initial levels, but systematically adds discriminant attention, response inhibition, cognitive flexibility, working memory and multiple simultaneous attention to constitute a general executive function training. The other games also demand sustained attention, but focus on use of categories, pattern recognition or spatial working memory. They use new algorithms to individualize moment-to-moment difficulty and progression through different task configurations. In addition, the overall BT includes physical exercise classes with both general aerobic exercise and exercises with cognitive components designed to activate the same neurocognitive systems as do the computer games. Data from thousands of children (some published^17^ and more available upon request) has consistently showed significant improvement on research tests of focused attention, response inhibition and working memory that are quite different from the games in content, format and user experience, indicating generalization of gains beyond the training games to the underlying cognitive operations. The present study was designed to evaluate immediate (cognitive priming) and longer-term effects (academic achievement) of BT on math and reading performance and learning. Can a BT game immediately before a math or reading curricular content game (CC) improve performance on the CC game (cognitive priming)? Does BT over several months increase performance on school-administered standardized tests of math and reading achievement beyond improvement in comparison classes that did not do the BT (cognitive enhancement and far transfer of BT effects to academic achievement)?

It is well established that prior stimulation can alter subsequent information processing. Presentation of a “priming” word can enhance recognition, classification or generation of words that are semantically related to the prime^18^. An alerting cue before stimulus presentation enhances response to the stimulus^19^, while a task-related visual stimulus delays response to immediately following stimuli in the same location in the visual field^20^. Cognitive operations activated by processing mathematical equations can facilitate subsequent language tasks^21^. Reading essays containing the individual “I” pronoun rather than the collective “we” pronoun prior to fMRI increases the greater right frontal activation associated with viewing self-photographs, an increase associated with faster reaction times^22^. Cognitive operations are supported by dynamically reconfiguring neural systems defined by the relative activation and interconnection of processing resources distributed throughout the brain. Task-set is a neural system configuration for performing the task. Operations priming^19^ as in the studies described above partially constitutes the task-set for the subsequent task.

Constituting an effective task-set is a pedagogic challenge in teaching young children. On the behavioral level this is evident in getting them to attend, persevere, comprehend and recall. Neurocognitively, executive function and associated brain resources essential for learning curricular material are still actively developing throughout school years. In addressing our first question, we sought to determine whether BT games designed to activate neural systems associated with executive function could prime these systems and thereby help constitute a neurocognitive task-set more conducive to learning math or reading. If so, it may then be possible to systematically determine what type of prime is generally best for what type of curricular content learning, and what kind of priming is best for each child.

Our second question relates to the more common use of BT to enhance neurocognitive function over a training period of several months. The proximal goal is to promote activity-dependent development of neural systems that support executive function. The key question is whether such gains can actually enable students to gain more from daily math and reading curriculum, and perform better on school-administered achievement tests of math or reading. If so, this would demonstrate far-transfer and real-world significance of BT, the existence of which is currently a contested issue.

## Results

### Question 1: Cognitive priming Effects on Reading and Math Performance

#### Reading CC Game

Accuracy on the Reading CC game was enhanced by priming with the Pattern Recognition (p < 0.001), General Executive Function (p < 0.001) and Spatial Working Memory (p = 0.01) BT games. When speed and accuracy are considered together, the positive effects of all four BT games are larger and significant (Categories p = 0.05, all others p < 0.0001). The Pattern Recognition game had the greatest effect and the Categories game the least effect, with the difference between the two approaching significance (p = 0.08) (Table 1).

#### Math CC Game

Accuracy on the Math CC game was significantly enhanced by priming with the Pattern Recognition BT game (p < 0.001), and the effects of the General Executive Function and Spatial Working Memory BT games approached significance (p = 0.07). The difference in effect between the Pattern Recognition and the Spatial Working Memory BT games approached significance (p = 0.095). When speed and accuracy are considered together, the positive effects of all four BT games are again larger and significant: General Executive Function p = 0.000001, Categories p < 0.000001, Pattern Recognition p = 0.001, and Spatial Working Memory p = 0.008. The Category BT game led to significantly greater performance enhancement than either the Spatial Working Memory (p = 0.016) or Pattern Recognition (p = 0.034) BT game (Table 2).

According to Keith’s rules for effects on school learning, effects between 0.05 and 0.10 are considered small but meaningful, between 0.10 to 0.25 are moderate, and above 0.25 are large^23^. Of the 16 priming effects evaluated, 11 are statistically significant and, using these guidelines, 8 of the 11 are of moderate effect size (Tables 1 and 2).

### Question 2: Far-Transfer to School-Administered Math and Reading Achievement Tests

**Figure 1:**
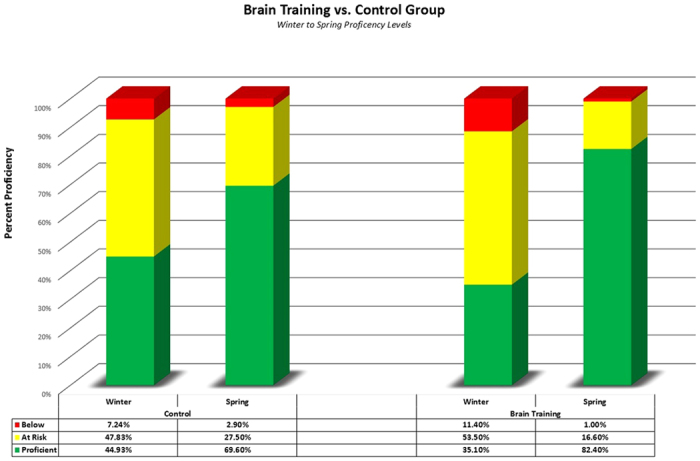
School-administered test of math achievement. Left-side bars are Winter and Spring scores of children in control classes that did not do the brain training. Right-side bars are Winter and Spring scores for classes that did the brain training between the two test dates. The school sets the cut-off scores for proficiency and below.

Program participants showed significantly greater gains than students in control classes that did not do the BT but took the same math and reading tests. The Group (BT vs. No BT) by Time interaction was significant for both math (F(1, 284) = 9.3, p < 0.001) and reading (F(1, 449) = 2.9, p = 0.04). Baseline scores (mean +/− sd) did not differ between the BT and control classes in either math (74.10 +/− 16.7 vs. 74.23 +/− 15.6) or reading (22.65 +/− 5.5 vs. 22.54 +/− 5.8). As indicated by the Group by Time interactions, the differences between groups were larger at post-test in both math (91.67 +/− 10.8 vs 86.25 +/− 12.4) and reading (29.92 +/− 6.2 vs. 29.18 +/− 4.8). The effect size (Cohen’s d) was 0.49 for math and 0.18 for reading. When subjects with perfect or near-perfect baseline scores are not excluded (see Methods), the effect on math remains significant at p < 0.001 but the effect on reading is not significant. The school system has performance cutoffs to classify students as proficient, at risk, or deficient as shown in Fig. 1 (green, yellow and red respectively) for math test scores with all students included. Despite starting the year with fewer children meeting proficiency, by year-end the brain-training class had 12.8% more children meeting proficiency than the control class. Evidence of even greater gains in proficiency in classes that used the BT program compared to district norms or same-school comparison classes have been provided by other schools for both math and reading, but without individual student data to allow statistical analysis (Supplemental data 1).

## Discussion

Doing a five-minute brain-activation video game before computerized math or reading curricular content learning games improved performance on the curricular content games. To put this into context, currently teachers try to establish mind/brain states (task-sets) suitable for learning by creating appropriate classroom environments (e.g., free of distraction, good lighting) and pedagogic techniques like “if your eyes are not on my eyes, you are not paying attention to me.” We provide proof of concept that short video games designed to activate specific neurocognitive processing systems can serve as brain warm-up calisthenics to improve cognitive performance immediately following the video game. Compared with procedures currently available to teachers, video-game cognitive priming can more directly establish internal states to facilitate learning, and make those states specific to the nature of the content material that follows. Moreover, technology readily allows the cognitive priming to adjust to possible differences in cognitive characteristics of different children. Applications are also possible in learning and performance situations other than schools.

Our data show different cognitive priming effects among the four BT games and as a function of the type of curricular content material that follows. Priming of math CC game performance was significantly greater following the Categories BT game than following either the Spatial Working Memory or Pattern Recognition BT games. In contrast, priming effects on reading CC game performance was the weakest with the Categories BT game and the greatest with Pattern Recognition BT game, with the difference between the two approaching statistical significance in the opposite direction from that with the reading CC game suggesting a double dissociation. This type of specificity is consistent with our assumptions that the different BT games activate non-identical neuro-processing systems, and that somewhat different task-sets are best for learning math and reading.

Our second finding is that children who played the BT games over a 12–16 week period showed greater improvement in school-administered tests of math and reading achievement than did control classrooms. The effect size on math outcomes (0.49) was greater than what is seen with one-on-one tutoring (0.40)^24^. This effect was achieved with one teacher and over 20 children rather than one teacher for each child, and led to improved reading outcomes at the same time while tutoring needs to be repeated for each subject area. The effects of both math (0.49) and reading (0.18) were substantially greater than what is seen with extended instruction in after school, weekend and summer programs^25^. The positive findings in this study in contrast to the absence of far-transfer in many previous studies in children are probably due to differences in the BT itself. The BT in this study engaged and trained a wide range of neurocognitive functions with the goal of using each as channels to activate neural systems that support executive function. This contrasts with the most often studied previous programs which narrowly focus on single dimensions of cognition^26^. Moreover, the current BT program uniquely combines plateau and graduation criteria to regulate movement between difficulty configurations in a way that powerfully individualizes training. Finally, the current BT included physical exercise as well as computerized exercise while previous programs have not. The computer-presented and physical exercises were designed to work synergistically, and while this may be one reason for the positive outcomes in the present study it also means that it is not possible from the current study to determine the relative contribution of each to observed outcomes. In addition, we note that the present study did not include an active control condition, therefore leaving open the possibility that some other non-specific aspects of the experimental condition such as relaxing the child or inducing positive mood could also have contributed to the positive outcomes. However, the fact that the effect sizes of our BT intervention are substantially greater than the effects of other school-based interventions that were also compared to “education-as-usual” is consistent with the BT having specific effects^25^.

Effects of BT were greater on math achievement than on reading achievement, but that may have more to do with the psychometric properties of the achievement tests than the nature of the BT. The math test was an objective paper and pencil test which may make it more reliable than the more subjectively scored and individually administered reading test. Moreover, data from the publishers of the reading test indicate limited inter-rater reliability of teacher scores, potentially adding to variance, and extremely high test-retest stability when teachers re-administer test suggesting that change scores may be constrained by stable preexisting impressions of student skills. In addition, the control groups for the math and reading outcomes were different. While not a problem for demonstrating the benefits of the BT on academic outcomes, it is when trying to compare the magnitude of effects on math and reading achievement.

The present study has limitations. First, the four types of BT games were similar in many ways and were designed to activate overlapping neural systems associated with EF. It is important that despite these similarities, they had significantly different priming effects, indicating that even within the narrow range of variability of the BT games used the particular nature of the priming activity affected the priming. But because of these similarities we have not defined the limits and essential features of an effective cognitive prime for math or reading. A second limitation is the absence of an active control in evaluating the far-transfer effects on the school-administered math and reading achievement tests. It is possible that enthusiasm about participation in a new school program carried over into greater motivation and effort when taking the achievement tests, the “Hawthorne Effect.” For several reasons, however, this is very unlikely to account for our findings. The computer exercises are highly repetitious, soon lost novelty or associated enthusiasm, and were just part of the school week. More importantly, for the group that did the BT + reading CC game there was no overt association with math and yet they showed very large gains on the math achievement test. The math achievement test was given at the end of the year along with other school tests and there was nothing to associate this test with the BT + reading CC games they had done. Similar considerations apply to reading outcomes in the group that did the BT + math CC games. Most important is a meta-analysis of 21 school-based intervention studies that all also used education-as-usual as a non-active control and broad math and/or reading achievement tests like the ones in our study as outcome measures. The mean effect size was 0.07^24^. If you assume that these interventions had no real effect at all, and attribute the entire effects to the Hawthorne effect, the effects we observed were seven times as great on math outcomes and over two and a half times as great in reading outcomes. Third, because the BT program requires a substantial commitment of curriculum time, the school system first identified schools willing to implement the program and then selected comparison schools that matched those schools in student demographics, staff stability, previous year test scores and overall resources. This has the disadvantage of not being random assignment, but has the advantage of ensuring that the experimental and comparison schools were as similar as possible. Since most activities each day in all schools aim to improve student performance there is no reason to think the experimental and comparison schools differed in motivation to succeed. Finally, using school-administered achievement tests to demonstrate far-transfer of BT has important advantages but psychometric limitations. Ceiling effects meant we were unable to assess the potential benefit of BT for high achieving students, and elimination of those students from analyses because they had little or no room to show effects of BT introduced an asymmetrical regression to the mean potentially artificially increasing average improvement in both BT and control groups. Such an effect could have reduced the difference between BT and control groups.

In conclusion, this study provides strong evidence of far-transfer of brain-training effects in children, an important and contested issue in the literature. In addition, this study provides the first data of which we are aware showing that a short brain warm-up or cognitive priming game just before an online curricular content game can facilitate performance on the curricular content game. This “proof-of-concept” opens the way for work to determine which type of cognitive priming of brain activation best facilitates which type of curricular content performance, how this varies among individual children and within children over time, and how this immediate priming can contribute to long term progress in curricular content learning. The study was done in a real-world school setting demonstrating that the BT and cognitive priming can potentially be widely used aspects of a neuroscience-based and technology-enabled pedagogy.

## Methods

### Participants

The program was instituted as part of the school day curriculum for 372 second graders in 13 classrooms in 4 Fairfax County Virginia public schools (FCPS). Classes within each school were randomly assigned to receive either BT plus the math CC content game (170 students) or BT plus the reading CC game (202 students). Three additional schools with 10 second-grade classes served as controls receiving neither the BT or CC games (72 students as controls for math outcomes and 139 for reading outcomes). Experimental and control schools were similar demographically, with 5–10% of children in all schools qualifying for free or reduced lunch based on low family income. All study procedures were approved by the Yale University School of Medicine Human Investigations Committee and all study procedures were in accordance with relevant guidelines.

#### Computer-Presented Brain Training (BT) Games

**Figure 2:**
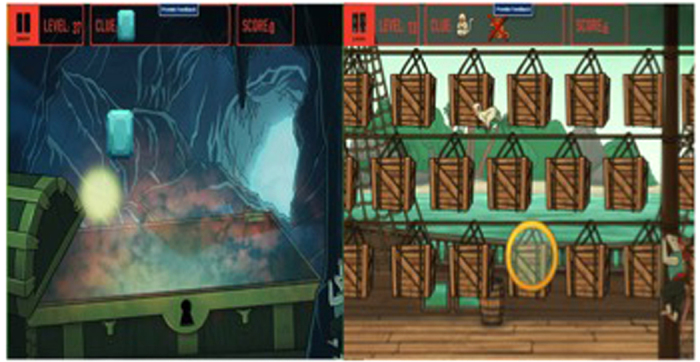
Two User Interfaces for the General Executive Function Brain Training Game. Left panel is the version where the child follows the moving light and clicks on it when it turns into a jewel that meets the criteria for being a target. There are two moving lights at the game level shown, and at the moment of the screen shot one has just turned to a blue jewel. The clue at the top of the screen shows that the target is blue jewels. The right panel is second user interface for the same cognitive demands and computer code. At the moment of the screen shot, the moving lens (yellow circle) has just revealed a target monkey in the crate, the child has clicked on it and monkey is freed from the crate and is running away. Computer code for the games was created with the text editor Sublime (https://www.sublimetext.com/) and art work created in Photoshop (http://www.photoshop.com/).

Game 1 was designed to train focused attention, response inhibition, cognitive flexibility but working memory and multiple simultaneous attention (**General Executive Function**). There are two versions of this game with the same underlying computer code and sequence of cognitive challenges, but a different user game experience (Fig. 2). In one version of this game, students begin by using the mouse to track a moving light and click on it when it turns into a red jewel. With correct responses, the light moves faster, with mistakes it slows down. As the game continues, more aspects of executive function are added. Blue jewels appear that should not be clicked, adding discriminant attention and response inhibition. Next, the target switches randomly between red and blue, increasing response inhibition demands and adding cognitive flexibility. Working memory is introduced by showing half-jewels and instructing children to only click on one that is the same color as the one before in order to create a full jewel. In addition to the primary focus on executive functions, this game also requires visual-spatial processing and hand-eye coordination. All levels repeat with two and three moving lights on the screen. A second version has the same underlying computer code and sequence of cognitive challenges, but a different user game experience (Fig. 2): a magic lens jumps from one moving crate to another revealing if there is a monkey inside. If it is a “target” monkey, the child clicks on it and the monkey runs out of the crate. We realize that the neuropsychological constructs of each component executive function (e.g., response inhibition) do not have a one-to-one correspondence with any operational aspect of game play (e.g., not responding to foils), as the operational behaviors require a mix of multiple functions that itself has still another overall identity in the moment on the neurophysiological level. But we do think that the progressive mix of operations across game levels, and the mix of operations across the set of BT games, constitute a BT program that intensively activates a set of neural functional systems that support executive functions. Game 2 trained use of categories (**Categories**). A pirate throws things from a chest into the air and children have to click on items in target categories before they fly off the screen. With correct responses they move more quickly, and progressive levels add up to six flying objects on the screen at once. Categories initially are simple and natural categories like letters, numbers, animals, plants, food and furniture. Categories on higher levels include tools, machines, sports and “things to take on a vacation.” At the highest levels, the child has to find objects on the screen that are in the same category. Game 3 trained pattern recognition (**Pattern Recognition**). Students see three objects in a row and have to choose one of three additional objects to complete the pattern. Ducks hold the objects and fly off happily when the row is successfully completed. The game begins with simple pattern rules like “all the same shape” or “all the same color”, and progresses to rules like “all different in color and shape” and patterns like “blue circle-yellow triangle –blue circle-yellow triangle.” Higher levels include number patterns, rotating shapes, and mixes of different kinds of problems. With correct responses, the time allowed for response becomes shorter, with mistakes it becomes longer. Game 4 trained spatial working memory (**Spatial Working Memory**). Children have to remember the order in which a group of pirates seated on the beach raise their hands to request dinner, or the places in a campsite visited by a playful monkey, clicking on them in appropriate order. The number of locations to be remembered begins with two, increases with success and decreases with mistakes. Some levels require the child to respond in reverse order. Game instructions are presented both aurally through headphones and visually on the screen.

#### Physical Brain-Training Exercises (PE)

Like the computer exercises, cognitive aspects of the physical exercises begin with sustained attention and response inhibition, and progressively layer in cognitive flexibility, multiple simultaneous attention and working memory. For example, initially children are each assigned their own space within their own circle on the floor, attend to their own bodies and practice yoga-like balancing poses. Next they do controlled ball passing in pairs, group running games with rules that require planning, strategy and self-control, or response inhibition games like “Simon Says.” Later they learn martial arts and dance sequences, or throw two different colored bags to one another in circles of 5–6 children, with each color having a different sequence of individuals to whom it is thrown. Each day there is a mix of more and less aerobic games, and group and individual focused exercises. While the physical exercises and computer exercises have highly similar cognitive demands, and were designed to activate similar, overlapping neural functional systems, we do not have brain imaging data to confirm the degree of overlap.

#### Curricular Content (CC) Games

Reading and math games were created for this study following New York State common core learning goals for second grade. For both the reading and math games we assessed two aspects of performance. First was accuracy, measured by correct responses/total responses, and independent of the speed of responses. The second measure, correct responses/minute, combined speed and accuracy. For example, if a child gets 100% accuracy but works very slowly, his correct responses/min will be quite low. A second child who gets 50% accuracy but works twice as fast will get the same score for correct responses/min. If two children respond equally fast, the one who makes a higher percentage of correct responses will get a higher correct responses/min score. Different individuals may prioritize different performance goals or strategies, and cognitive priming may differentially impact the strategies and goals reflected in these two measures. Locating each individual in the two-dimensional space of these measures and performance dimensions, and assessment of how the relative prioritization may change over time, are beyond the scope of the current report. However, the two measures allow us to begin to consider how different kinds of priming may impact different performance goals and strategies in the group as a whole.

### Reading Game

The reading game requires children to make word chains with correct links defined by similarity of vowel sounds, although the game relies only on visual stimuli and matching. Chains anchor floating pirate ships, and a small pirate swims under water to place words selected from options in a fishing net onto the chain. Over the 31 game difficulty levels, matches go from simple to more complex (e.g., pat-hat-sat-mat to day-say-sunday-sundae-hay-hey-sway-play-grey-weigh-neigh-stray).

### Math Game

The math game is based on Number Bonds - sets of three circles connected by lines, with a large circle containing a “whole” (e.g., 12) and the smaller circles containing its “parts” (e.g. 5 and 7). In the game this visual structure is presented as a balance scale. For addition, there are two smaller spheres on one side of the central balance point and a larger sphere on the other side. For subtraction, there are two spheres of equal size, one on each side of the balance. The child has to add or subtract gold from a specific sphere to balance the scale, choosing from a collection of gold pieces of varying value to move into a sphere in addition, and choosing which gold pieces to remove from a sphere in subtraction. The problems are designed to increase understanding of numbers (numeracy) and concepts of 10’s and 100’s, and increase comfort adding and subtracting two and three digit numbers. Problems become more difficult as students progress through 120 game levels. In calculating performance measures for the math game, moves that balanced the scale were considered correct moves. In this game, moves that precede the move that actually achieves balance are not right or wrong but excessive moves accumulate to lower scores. For example, if the problem 6 + ? = 24 with 6 in one sphere on the left and nothing in the second sphere on the left, can be answered more quickly by adding one 10-unit piece and two 4-unit pieces than by adding a 5-unit piece, three 2-unit pieces and 1-unit pieces until the scale balances. The first strategy will yield a higher accuracy score (1/3 vs. 1/11), and potentially a higher combined speed and accuracy score since the number of scales balanced/min reflects the number of moves needed to achieve balance and the speed of each move.

### Performance and Academic Outcomes

Far transfer of BT was evaluated by change in school-administered standardized tests of math and reading achievement given to participants just before beginning (pre-test) and upon completion (post-test) of the program, and to controls at the same time points. The math achievement test was developed by FCPS and is used standardly throughout the district. It consists of 20 questions covering multiple areas of math-related knowledge that require students to identify symmetrical figures, identify and describe geometric shapes, use data to predict outcomes of experiments and construct graphs, identify patterns of figures, estimate and measure length, and use money, clocks and calendars, as well as numeracy, place value, arithmetic and fractions. Reading achievement was assessed with the Developmental Reading Assessment (DRA), 2^nd^ Edition of reading fluency and comprehension done by the teacher of each child individually, based on how the child reads aloud and understands standardized test passages. Instructional booklets prepare teachers for administering and scoring the test^27^. Test-retest reliability based on the same teacher rating the same students (n = 112, grades 1–6) with a 14-day interval (number of different teachers is not specified) is very high 0.97 (fluency) and 0.99 (comprehension). To assess inter-rater reliability, 26 raters evaluated audio recordings and reviewed student written work from 30 DRA administrations. Gwel’s Kappa First order agreement coefficient was 0.57 for fluency and 0.65 for comprehension, with 41−0.60 considered moderate and above 0.60 considered substantial agreement^27^.

### Brain Training Schedule

Children did the BT and CC computer games three or four times per week from February to June 2015, with a mean of 31 sessions per child, range of 6–65 with two outliers removed, and 81% of children doing at least 20 sessions. Sessions initially included five 5-min blocks of BT games and one 10-min CC game, but the CC game was reduced to 5-min approximately 20% into the study to accommodate school scheduling constraints. To evaluate potential cognitive priming effects, we compared CC performance when it was not preceded by a BT game to performance when it followed each of the four BT games. In order to minimize the number of days when class time ended before the CC game was played, the CC game was randomly presented as the first, second or third game of the day. Since we have four types of BT games, we have five conditions of interest (**CC** first, BT_1_/**CC**, BT_2_/**CC,** BT_3_/**CC** and BT_4_/**CC)**. Within each successive block of 10 training sessions, the two sessions in which the CC came first were selected randomly. In order to increase engagement, before children play a BT game, they see a “game-choice” screen allowing them to choose one of two BT games to play next. Their choices constrain future options within each 10-session randomization block so that each of the 4 types of BT games precedes the CC two times within the block. Children did the PE training games one or two times per week for approximately 30 minutes each day. Online materials at a teacher-portal provided tips for facilitating the computer exercise sessions and detailed daily lesion plans for the PE. Teachers received a 4-hour training in administration of the computer and PE training games and all sessions were led by the schoolteachers.

### Statistical Analyses

#### Immediate Cognitive Priming Effects on Math and Reading Performance

Unprocessed data from every key stroke or mouse click from every game was sent to the statistical analysis team at The National Center for Research on Evaluation, Standards, and Student Testing, CRESST/UCLA for analysis. Thus data analysis was done largely independently of researchers who designed this study of BT, and of the commercial company (C8Sciences) that supports the use of the program in schools, although the whole research team participated in discussion of the general goals and strategy of the analysis and interpretation of the findings. Evaluation of the effects of the BT games on CC game performance is statistically complex for several reasons. The structure of the data set is multi-level with game-play sessions nested within students. There are a number of potential covariates that may influence performance on the CC games. In addition only a subset of CC game levels are engaged in any given session. Thus expected session performance is also complicated by the properties of those levels that were engaged, which vary from one session to another. In order to account for these complexities we employ a flexible multilevel item response theory (IRT)^28^,^29^,^30^,^31^ approach with covariates to simultaneously model the variation within and between students, and also address the varying characteristics of CC game-play including CC game-play level difficulty.

The data set was screened for anomalous and outlier values. Sessions that were incomplete or overly long were eliminated, and if time spent on a particular game level within a session was less than one minute or less than 10 game moves, that data was not considered. These filters created a data set for analysis consisting of 5876 sessions (from 6,534 before the filters) of the reading CC game and 3884 (from 4,541 before the filters) of the math CC game. Chi-Square and RMSEA item fit statistics indicated excellent fit for all outcome measures. Reported p-values are 2-tail.

#### Far Transfer to School-Administered Math and Reading Achievement Tests

Differences between program participants and controls in improvement were evaluated by repeated-measures analysis of variance (ANOVA) run in R. Due to teacher error, 73 students in the BT group were given the wrong math test or no math test in the spring. This led to an analysis sample of 299 children who did BT for math outcomes and 372 for reading. The highest score observed on the reading tests was 38. The mean improvement from pre- to post-test in the entire sample of participant and control children was over six. In order to limit ceiling effects when evaluating possible differential improvement in participant and control groups, 53 children with pre-test scores of 34 or more were excluded from the BT group and 7 from the control group, leaving groups of 319 and 132. Children with pre-test scores of 90 and above (68 in the BT group and 24 controls) were excluded from analysis of math achievement where the top possible score was 100 and the mean improvement in the overall sample was over 16, leaving groups of 231 and 48. Analyses were repeated with no students excluded. One-tail tests were used since data from multiple previous school implementations showed class average increases in school-administered math and reading tests (Supplementary data 1). Distribution of pre/post difference-scores used in each analysis in each group met normality assumptions and the groups did not differ in variance of difference-scores by Fligner test.

## Additional Information

**How to cite this article**: Wexler, B. E. *et al*. Cognitive Priming and Cognitive Training: Immediate and Far Transfer to Academic Skills in Children. *Sci. Rep.* **6**, 32859; doi: 10.1038/srep32859 (2016).

## Supplementary Material

Supplementary Information

## Figures and Tables

**Table 1:** Cognitive Priming Effects (Standard Errors) on Reading Performance.

Cognitive Priming Game	Accuracy	Speed and Accuracy
General Executive Function	0.105 (0.029) p < 0.001	0.204 (0.049) p < 0.0001
Categories	0.051 (0.029) p = 0.078	0.099 (0.051) p = 0.052
Pattern Recognition	0.098 (0.029) p < 0.001	0.227 (0.053) p < 0.0001
Spatial Working Memory	0.080 (0.031) p = 0.010	0.185 (0.046) p < 0.0001

**Table 2:** Cognitive Priming Effects (Standard Errors) on Math Performance.

Cognitive Priming Game	Accuracy	Speed and Accuracy
General Executive Function	0.108 (0.060) p = 0.072	0.184 (0.038) p = 0.000001
Categories	0.101 (0.056) p = 0.071	0.223 (0.036) p < 0.000001
Pattern Recognition	0.205 (0.057) p < 0.001	0.115 (0.036) p = 0.001
Spatial Working Memory	0.067 (0.060) p = 0.26	0.099 (0.037) p = 0.008
