# Supplementary material for: Cognitive Priming and Cognitive Training: Immediate and Far Transfer to Academic Skills in Children
Source: Sci Rep. 2016 Sep 12;6:32859. doi: 10.1038/srep32859 (PMC5018694; doi:10.1038/srep32859)
Supplement: Supplementary Information [file srep32859-s1.doc]

Supplementary Information

Cognitive Priming and Cognitive Training:

Immediate and Far Transfer to Academic Skills in Children

Bruce E. Wexler^1^, Markus Iseli^2^, Seth Leon^2^, William Zaggle, Cynthia Rush^3^, Annette Goodman, A., Esat Imal^1^, Emily Bo^2^

^1^Department of Psychiatry Yale University School of Medicine, ^2^National Center for Research on Evaluation, Standards, and Student Testing, CRESST / UCLA, ^3^Department of Statistics, Yale University

**SREP-16-11950-T**

## 1 Supplementary Information

2

## 3 Supplementary Data 1

Pearson Math Assessment First grade, 95% Free Lunch

120

100

|  | 25 |  | 16 |  | 45 |  | 33 |  | 29 | 4  4 | | |
| --- | --- | --- | --- | --- | --- | --- | --- | --- | --- | --- | --- | --- |
|  |  |  |  |  |  |  |  |  |  |  | 92 |  |
|  |  |  | 21 |  |  |  |  |  |  |  |  |  |
|  |  |  |  |  |  |  |  |  |  |  |  |  |
|  | 30 |  |  |  |  |  |  |  |  |  |  |  |
|  |  |  |  |  |  |  |  |  | 52 |  |  |  |
|  |  |  |  |  |  |  | 15 |  |  |  |  |  |
|  |  |  | 63 |  |  |  |  |  |  |  |  |  |
|  |  |  |  |  |  |  |  |  |  |  |  |  |
|  |  |  |  |  | 14 |  |  |  |  |  |  |  |
|  |  |  |  |  |  |  | 52 |  |  |  |  |  |
|  | 45 |  |  |  |  |  |  |  |  |  |  |  |
|  |  |  |  |  | 41 |  |  |  |  |  |  |  |
|  |  |  |  |  |  |  |  |  | 19 |  |  |  |

80

60

40

20

0


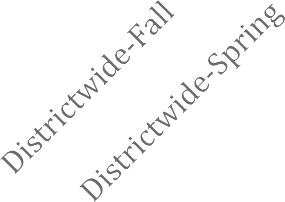

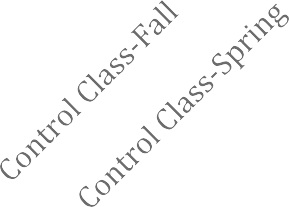

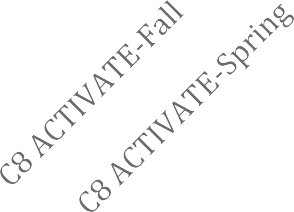


Pro@icient At Risk Below Pro@icient

4

5

120

# Pearson Reading Assessment 3rd grade

100

19

18

16

11

6

20

24

26

83

61

58

58

80

60

40

20

0


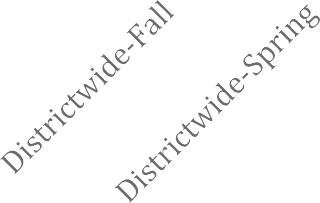

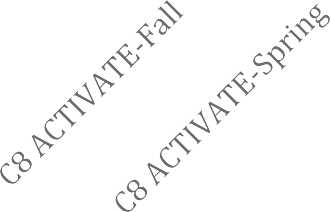


Pro@icient At Risk Below Pro@icient

6

7

120

# Pearson Math Achievement 2nd grade

Class Recieved Special Reading Program Class Recieved Activate

100

|  | 11.4 |  | 21.6 |  | 16.6 |  | 14.7 |  | 16.6 |  | 6.8 |  |
| --- | --- | --- | --- | --- | --- | --- | --- | --- | --- | --- | --- | --- |
|  |  |  |  |  |  |  |  |  |  |  | 20.6 |  |
|  | 26.8 |  |  |  |  |  |  |  |  |  |  |  |
|  |  |  |  |  |  |  | 22.1 |  |  |  |  |  |
|  |  |  |  |  | 29.1 |  |  |  | 26.6 |  |  |  |
|  |  |  |  |  |  |  |  |  |  |  |  |  |
|  |  |  | 26 |  |  |  |  |  |  |  |  |  |
|  |  |  |  |  |  |  |  |  |  |  | 72.3 |  |
|  |  |  |  |  |  |  | 63 |  |  |  |  |  |
|  | 61.4 |  |  |  |  |  |  |  |  |  |  |  |
|  |  |  |  |  |  |  |  |  | 56.6 |  |  |  |
|  |  |  |  |  | 54.1 |  |  |  |  |  |  |  |
|  |  |  | 52.1 |  |  |  |  |  |  |  |  |  |
|  |  |  |  |  |  |  |  |  |  |  |  |  |
|  |  |  |  |  |  |  |  |  |  |  |  |  |

80

60

40

20

0

Fall Winter Spring Fall Winter Spring

Pro@icient At Risk Below Pro@icient

8

1. In this school one class was given a special reading enhancement program and the
2. other was given our brain-­‐training program. The class that did the brain training
3. had non-­‐significantly greater gains in reading than did the class that got the special
4. reading program. Shown here, the class that got our brain training showed much
5. greater gains in math. N.B., this is comparison to an active control. 14

15

16

17

18

19

20

21

22

23

24

25

26

27

## 28 Supplementary Methods 1

29

## CC Reading Game Response Outcomes

1. We use Samejima’s graded response model^1^. In IRT graded response models the
2. response variables must be coded in such a way that each item has a response that falls
3. along an ordered scale with each value on the scale being present for each item. In this
4. study we treat the levels of CC game-play as if they were items on an assessment. For
5. ease of interpretation we keep the number of ordered response categories within each of
6. the two CC games consistent for all levels/items. The degree to which the values of the
7. performance constructs vary across levels is different in the CC math game as compared
8. to the CC reading game. As a consequence, the recoding of the continuous performance
9. constructs requires differing approaches that are described for each game in the
10. supplemental materials.

41

1. In the CC reading game there were 31 levels through which the students played. Students
2. received a continuous score on each of the three performance constructs (speed, accuracy,
3. and combined speed and accuracy). The continuous constructs were then binned into
4. quintiles based on performance across all the levels. This categorization resulted in score
5. coverage for all 5 categories in each of the 31 game levels (see Table 1). Setting the same
6. cut points for all 31 items has the benefit of easing the comparative interpretation of the
7. IRT level parameters with respect to level difficulty since the same scoring rubric applies
8. at each level.

50

51

## Table 1. Cut-Points for CC Reading Game Response Variables

|  | Speed- Correct Moves Per Minute | Accuracy - % Correct Moves | Standardized Speed and Accuracy |
| --- | --- | --- | --- |
| All Levels |  |  |  |
| Cut point 1 | <4.34 | <42% | <-0.69 |
| Cut point 2 | 4.34 to 6.69 | 42% to 54% | -0.69 to -0.22 |
| Cut point 3 | 6.70 to 8.45 | 55% to 67% | -0.22 to 0.19 |
| Cut point 4 | 8.45 to 10.43 | 67% to 79% | 0.19 to 0.66 |
| Cut point 5 | >10.43 | >79% | >0.66 |

1. **CC Math Game Response Outcomes**
2. In the CC math game there were 121 levels. The maximum number of levels engaged by
3. any student was 115 levels. In order to have adequate sample size coverage for IRT
4. analysis it was necessary to substantially reduce the number of levels by collapsing levels
5. with similar game-play characteristics. For example the original game levels one, three,
6. four and five all shared the same game-play configuration and problem type (addition
7. problem with a sum lower than five). These four original levels were recoded into a
8. single collapsed level. Following this process we were able to reduce the original levels
9. to a workable number of twenty-four collapsed levels.

62

1. In the process of attempting to create binned quintiles from the twenty-four collapsed
2. levels there still remained unfilled cells. This lack of adequate sample coverage suggested
3. that the game-play performance in the CC math game decreased more steeply across
4. game levels as compared to the CC reading game. Due to this performance decrease and
5. in order to ensure adequate sample coverage across all twenty-four collapsed levels it was
6. necessary to employ three ordered response categories in the CC math game in
7. comparison to the five categories that were used for the CC reading game.

70

1. There was also a sharp decrease in performance beginning with collapsed level number
2. eleven which had the game-play characteristic of adding ten to multiples of ten through
3. ninety. As a result it was necessary to set separate cut points for collapsed levels one
4. through 10 and eleven through twenty four. The resulting cut points for the three
5. response constructs are shown in Table 2. Interpretation of the IRT level difficulty
6. parameters should take these cut points into account.

77

## 78 Table 2. Cut-Points for CC Math Game Response Variables

|  | Speed- Correct Moves Per Minute | Accuracy - % Correct Moves | Standardized Speed and Accuracy |
| --- | --- | --- | --- |
| Levels 1-10 |  |  |  |
| Cut point 1 | <3.44 | <31% | <0.15 |
| Cut point 2 | 3.44 to 5.51 | 31% to 48% | 0.15 to 1.07 |
| Cut point 3 | >5.52 | >48% | >1.07 |
| Levels 11-24 |  |  |  |
| Cut point 1 | <1.92 | <10% | <-0.75 |
| Cut point 2 | 1.92 to 2.99 | 10% to 16% | -0.75 to -0.36 |
| Cut point 3 | >2.99 | >16% | >-0.36 |

79

1. The structure of our data set is one that is commonly seen in multi-level growth models.
2. Each student participated in multiple game-play sessions resulting in basic levels (or
3. dimensions) in the data set. Model level I represents the repeated sessions that are nested
4. within students thus capturing the within-student variation, and model level II captures
5. the between-student variation. We use the flexMIRT software^2^, which implements the
6. Metropolis-Hastings Robbins-Monro (MH-RM) algorithm^3-5^ which allows for the
7. estimation of higher dimensional models and which provides the capacity to combine
8. IRT with multi-level models that include covariates. In addition to generating game-play
9. level parameters, the IRT model also generates a latent factor for each level of the model.
10. We fix the variance of our between-student factor to one and allow the within-student
11. factor to vary freely. We also include eight covariates in our model, seven of which
12. predict the within-student (model level I) factor of our model and one which predicts the
13. between-student (model level II) factor.

93

1. The primary research question involves the immediate effect of four BT games on the
2. within-student CC game performance variation. These four treatment-related covariates
3. are coded as dummy variables and the condition with no BT games serves as a reference
4. group. The between-student latent variable variance was fixed at one and has a mean of
5. 0, so the estimate of the BT game dummy variable is in the format of an effect size. Three
6. other covariates are included in the model to predict the within-student factor (model

100

level I): 1) a simple linear indicator of the session day, which represents the current

101

session day for a student; 2) the length of time of each session; and 3), a variable that

102

indicates if the student had previously experienced any of the levels they engaged in a

103

current session. Finally, we also include gender as a covariate to predict the between-

104

student factor (model level II).

105

1. Kim, S. & Camilli, G. An item response theory approach to longitudinal analysis

106

with application to summer setback in preschool language/literacy. *Large-scale*

107

*assessments in Education*. **2,** doi: 10.1186/2196-0739-2-1 (2014).

108

1. Samejima, F. Estimation of Latent Ability Using a Response Pattern of Graded

109

Scores. *Psychometric Monograph No. 17*, Psychometric Society, Richmond, VA.

110

(1969).

111

1. Houts, C. R., & Cai, L. flexMIRT: Flexible Multilevel Item Factor Analysis and

112

Test Scoring *User’s Manual* (2012).

113

1. Cai, L. High-dimensional exploratory item factor analysis by a Metropolis-

114

Hastings Robbins-Monro algorithm. *Psychometrika*, **75**, 33– 57 (2010).

115

1. Cai, L. Metropolis-Hastings Robbins-Monro algorithm for confirmatory item

116

factor analysis. *Journal of Educational and Behavioral Statistics*, **35**, 307–335

117

118

(2010).
